# The Natural Progression of Gambiense Sleeping Sickness: What Is the Evidence?

**DOI:** 10.1371/journal.pntd.0000303

**Published:** 2008-12-23

**Authors:** Francesco Checchi, João A. N. Filipe, Michael P. Barrett, Daniel Chandramohan

**Affiliations:** 1 Department of Infectious and Tropical Diseases, London School of Hygiene and Tropical Medicine, London, United Kingdom; 2 Department of Plant Sciences, University of Cambridge, Cambridge, United Kingdom; 3 Division of Infection and Immunity, Institute of Biomedical and Life Sciences, Glasgow Biomedical Research Centre, University of Glasgow, Glasgow, United Kingdom; University of Edinburgh, United Kingdom

## Abstract

Gambiense human African trypanosomiasis (HAT, sleeping sickness) is widely assumed to be 100% pathogenic and fatal. However, reports to the contrary exist, and human trypano-tolerance has been postulated. Furthermore, there is uncertainty about the actual duration of both stage 1 and stage 2 infection, particularly with respect to how long a patient remains infectious. Understanding such basic parameters of HAT infection is essential for optimising control strategies based on case detection. We considered the potential existence and relevance of human trypano-tolerance, and explored the duration of infectiousness, through a review of published evidence on the natural progression of gambiense HAT in the absence of treatment, and biological considerations. Published reports indicate that most gambiense HAT cases are fatal if untreated. Self-resolving and asymptomatic chronic infections probably constitute a minority if they do indeed exist. Chronic carriage, however, deserves further study, as it could seed renewed epidemics after control programmes cease.

## Introduction

Human African trypanosomiasis (HAT, sleeping sickness) due to *Trypanosoma brucei gambiense* is a disease of long duration. However, our understanding of the natural progression of gambiense HAT infection in the absence of treatment remains surprisingly poor. Parasites become patent in the blood within 1–2 weeks of infection, heralding the haemolymphatic stage (stage 1), which is characterised by non-specific signs and symptoms. Eventually, parasites penetrate the blood–brain barrier (BBB), leading to the meningoencephalitic stage (stage 2), which features more specific signs and neurological symptoms, and leads to coma and death.

**Figure 1:**
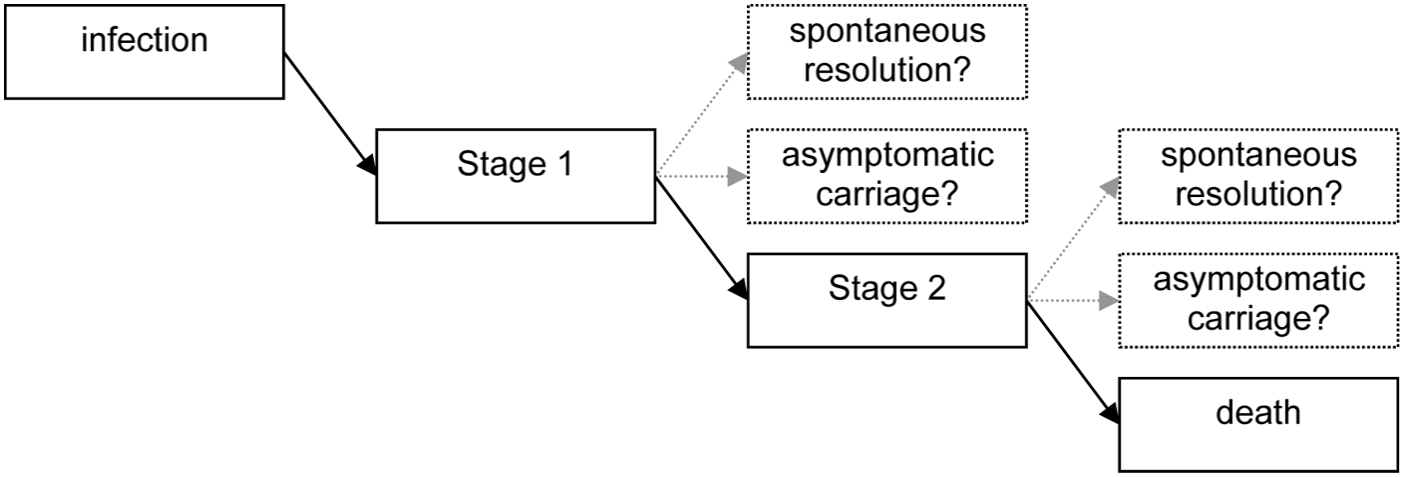
Possible Outcomes of Untreated Gambiense Human African Trypanosomiasis.

A commonly held assumption is that HAT always [1] or almost always [2],[3],[4] progresses to stage 2, and is always fatal if untreated [4],[5]. However, the empirical evidence to support this assumption is limited, and, at least theoretically, four “trypano-tolerant” alternative outcomes are possible: (i) early (i.e., within weeks or months) spontaneous resolution of stage 1; (ii) chronic, asymptomatic, or mildly symptomatic carriage in stage 1 without progressing to stage 2; (iii) progression to stage 2 followed by early spontaneous resolution; and (iv) chronic, asymptomatic, or mildly symptomatic carriage in stage 2 (Figure 1).

There is uncertainty about the average duration of stage 1 and stage 2 pathogenic gambiense infections in the absence of treatment, and its degree of variation across cases. Stage 1 has been reported to last “many months, sometimes over two years” [6] or “several months to two years” [5]. The duration of its asymptomatic phase may be many months if not years [2],[5], and this is followed by “a few months” of a non-specific syndrome [7]. Sir Patrick Manson wrote that disease may start as late as 7 years after HAT infection [4]. After the onset of stage 2, death occurs “within a few months, but may be delayed for up to a year” [6], “from four months to one year” [5], or from “four to eight months, exceptionally beyond one year”, according to Manson [4]. There is probably a systematic delay in the detection of stage 2, since parasites may take some time to travel from the brain to the lumbar region, where punctures to collect cerebrospinal fluid (CSF) for diagnostic purposes are usually taken for reasons of safety. The extent of this delay is not known. Overall, the entire duration of the infection may be “from one to three years after onset” of initial symptoms [8], or “2–5 years” [9].

Gambiense HAT control is largely based on case detection and treatment. This reduces the duration of infectiousness and thus diminishes onward transmission and the effective reproductive ratio (the average number of secondary cases arising from one case). While active case detection through mass screening campaigns is associated with substantial reductions in HAT transmission, total elimination (defined here as zero transmission) has proved hard to achieve. For example, historical foci in Cameroon [10] and the Ivory Coast [11] have survived at low prevalence despite years of case detection. Numerous hypotheses have been advanced to explain this extraordinary persistence. These include constant reseeding with infections from outside the focus; the role of a putative animal reservoir; and the existence of chronic, asymptomatic carriers, or cases of extremely long infection, which maintain low-level transmission and, once control is relaxed, may lead to a new outbreak. Indeed, the epidemiological dynamics of a pool of undetected chronic carriers would be similar to that of an animal reservoir, already shown theoretically to reduce the transmission impact of case detection [12].

Clearly, the latter hypothesis can only be addressed if one tackles fundamental unknowns. For example, do some HAT infections progress to non-pathogenic outcomes, and if so, with what frequency? What is the range of duration of untreated infections? The potential impact of interventions to reduce HAT transmission through prompt case detection is greater the longer cases remain infectious in the absence of treatment. Furthermore, if asymptomatic (or benignly symptomatic) chronic carriers do make up a significant proportion of infected people, control strategies based on passive case detection of ill patients might never be sufficient to eliminate HAT, and active case detection, with treatment irrespective of symptoms, would be required to interrupt transmission. On the other hand, exposing asymptomatic cases who will not progress to disease to the toxic and cumbersome HAT drugs currently available would entail an unnecessary risk of side effects.

Better evidence on the above issues would be directly useful for clinicians and HAT control programmes. Moreover, the long-term strategy to control and possibly eliminate HAT would benefit from quantitative predictions of the impact on transmission and mortality of different HAT control strategies, and of the duration and intensity of control needed to achieve set targets. Mathematical models can produce such predictions and have guided policy decisions for various tropical diseases, including onchocerciasis [13] and schistosomiasis [14]. However, these models are misleading if they are based on inaccurate assumptions about the basic parameters of the infection in hosts and vectors. The present paper aims to improve current evidence on some of the key human parameters, with a view to developing more realistic and thus useful models of HAT transmission.

We recently derived an indirect estimate of the duration of untreated pathogenic stages 1 and 2 HAT [15]. Here, we present a literature review of the duration and evolution of *T .b. gambiense* infections in the absence of treatment, and biological considerations about the parasite. Throughout this paper, “HAT” refers to gambiense infection.

## Literature Review

### Search Strategy and Results

A PubMed MeSH search of Medline references under the general term “Trypanosomiasis, African”, covering the period 1953–2006, was performed. We also did simple PubMed searches with all possible combinations of the terms “healthy carrier”, “disease progression”, “duration”, “latency”, “tolerant”, or “tolerance” with the terms “sleeping sickness”, “trypanosome”, “trypanosomiasis”, or “trypanosomosis”, as well as the French, Spanish, Portuguese, and German terms for “trypanosomiasis” and “sleeping sickness”. We screened resulting references for articles concerned with in vivo human infections, excluding general clinical reviews. We also screened all African trypanosomiasis abstracts contained in the *Tropical Disease Bulletin* (a comprehensive abstract book for tropical medicine, covering journals published in English, French, Spanish, Portuguese, German, and Italian) from 1910 until 1952. We followed the bibliographic trail of each relevant citation backwards in time until its exhaustion. We also reviewed all meeting reports of the International Scientific Committee for Trypanosomiasis Research, and contacted ten leading HAT experts. We generally limited our search to reports published since 1910; before that time, HAT parasitology and diagnosis, as well as scientific journals, were in their infancy.

We focussed on three types of report. First, we identified reports of the natural progression of infection in untreated patients whose status at baseline and at the end of observation was known, and we only retained those in which patients' diagnosis was confirmed microscopically or by the polymerase chain reaction (PCR). Second, we identified reports of the duration of stage 1, stage 2, or both in untreated patients who did progress to disease or death, and here we included patients irrespective of whether diagnosis had been confirmed parasitologically. Third, we identified reports of infections confirmed by microscopy or PCR at baseline that had cleared (at least three consecutive negative blood samples) without treatment at the end of the observation period.

The search yielded 27 eligible reports of the natural progression of gambiense infection, 42 eligible reports of the duration of infections progressing to disease, and four reports of spontaneously cleared infections. We also found three reviews [16],[17],[18]. One reference could not be retrieved [19].

### General Quality of Reports

Most reports contained little detail on study methods, and few relied on substantial sample sizes (Table 1 and Table 2). Only some studies began observing patients at the time of infection and/or followed them until death or cure. Most reports listed patient status at baseline or end of follow-up simply as “stage 1” or “stage 2”, without information about time elapsed since infection or stage 2 progression. Some studies included a mix of stage 1 and 2 patients.

**Table 1:** Summary Findings from Eligible Reports about the Natural Progression of Untreated Gambiense HAT Cases, Ranked by Duration of Observation Period

Author	Year of Patients' Diagnosis	Modern-Day Country of Infection	Method of Diagnosis^a^,^b^	Type of Case Detection^c^	Number of Patients	Years and Months under Observation	Outcome (*n*)			CFR (%)^b^
							Dead	Alive (*n* Healthy if Known)	Unknown or Disappeared	
Lester [76]	1929–1931	Nigeria	DM	A	≈2700	1 m	80	?	?	≈3
Duggan [77]	1940s	Nigeria	DM	A	?	1–2 m	?	?	?	≈2
Harding & Hutchinson [33]	1944–1945	Sierra Leone	DM	A	75	2 m	0	75 (75)	0	0
Harding [78]	1934	Nigeria	DM	A	≈400	3 m	20	?	?	≈5
Van Hoof [79]	1940s	DRC^d^	DM	A	12	6 m	0	12 (12)	0	0
Marshall & Vassallo [26]	1921	Uganda	DM	A	123	6 m	18	90	15	15
Marshall & Vassallo [26]	1921	Uganda	DM	A	118	7 m	25	73	20	21
Harding & Hutchinson [33]	1944–1945	Sierra Leone	DM	A	17	7–9 m	0	14 (13)	0	0
Jamot [80]	1920s	Cameroon (“epidemic”)	DM	A	?	1 y	?	?	?	50–70
Jamot [80]	1920s	Cameroon (“endemic”)	DM	A	?	1 y	?	?	?	25–30
Marshall & Vassallo [26]	1921	Uganda	DM	A	40	1 y and 6 m	20	12 (≤6)	8	50
Marshall & Vassallo [26]	1921	Uganda	DM	A	28	1 y and 6 m	9	11	8	32
Marshall & Vassallo [26]	1921	Uganda	DM	A	26	1 y and 8 m	16	0	10	62
Wade [81]	1911–1912	Ghana	DM	C	32	1–2 y	?	≥9	?	?
Wade [81]	1910	Ghana	DM	C	97	3 y	?	≥20	?	?
Kleine (in Yorke [17])	1911	Cameroon	DM	C	565	3 y	?	?	?	51
Todd (in Yorke [17])	1903	DRC	DM	C	102	3 y	?	34	?	?
Barlovatz [35]	1929	DRC	CM	A	14	3 y and 3 m	6	8 (8)	0	43
Woodruff et al. [82]; also in Taelman et al. [83]	1981	DRC	PCR	V	1	3 y and 3 m	0	1 (0)	0	
Jamonneau et al. [28]	1995–1996	Ivory Coast	CM, PCR	A	15	3–4 y	0	15 (11)	0	0
Heckenroth [27]	1907	DRC	DM	C	36	4 y	21	6 (3)	8	60
Greggio [25]	1911	DRC	DM	A	33	4 y and 6 m	24	9	0	73
Ringenbach [84]	1907	Republic of Congo	DM	C	1	5 y	0	1	0	
Méda & Doua (in Pépin & Méda [42])	Unknown	Ivory Coast	CM	Unknown	5	3–6 y	0	5 (0)	0	
Jamonneau et al. [29]	1995	Ivory Coast	CM, PCR	A	6 (subset of [28])	7 y	0	6 (3)	0	
Todd [30]	1911	Gambia	DM	C	12	9 y	1	8	0	8
Todd [31]	1911	Gambia	DM	C	1 (subset of [30])	13 y	0	1	0	

a DM  =  direct microscopy on blood, cerebrospinal fluid, or gland puncture fluid; CM  =  microscopy after blood concentration; PCR  =  polymerase chain reaction.

b Case fatality ratio; only calculated if number of patients under observation >10.

c A  =  active community screening; C  =  convenience screening; P  =  passive case detection; V  =  vertical transmission of HAT to patient's baby.

d Democratic Republic of the Congo.

**Table 2:** Summary Findings from Eligible Reports about the Duration of Untreated Stage 1 or 2 Gambiense HAT

Author	Year of Patients' Diagnosis	Modern-Day Country of Infection	Method of Diagnosis^a^	Type of Case Detection^b^	Number of Patients	Period of observation		Years and Months to Outcome
						From	To	
Kerandel [85]	1907	Republic of Congo	DM	P	1	Infection	Stage 1, ill^c^	4 m
Low & Manson-Bahr [86]	1922	Nigeria	DM	P	2	Infection	Stage 1, ill c	3 m; <1 y
Stephens & Yorke [87]	1922	Nigeria	CM	P	1	Infection	Stage 1, ill c	7 m
Cooke et al. [36]	1936	Nigeria	DM	P	1	Infection	Stage 1, ill c	4 m
Crastnopol et al. [88]	1962	Sudan	DM	P	1	Infection	Stage 1, ill c	3 m
Coulaud et al. [89]	1973	Gabon	CM	P	1	Infection	Stage 1, ill c	<9 m
Taelman et al. [83]	1982	DRC	CM	P	1	Infection	Stage 1, ill c	≥7 y
Scott et al. [90]	1990	Nigeria or Gabon	DM	P	1	Infection	Stage 1, ill c	<3 y
Nattan-Larrier & Ringenbach [91]	1911	Republic of Congo	DM	P	1	Infection	Stage 2	<2 y and 5 m
Ortholan [92]	1911	Republic of Congo	DM	P	1	Infection	Stage 2	1 y to 4 y
Sicé & Leger [93]	1920s	Various	DM	P	6	Infection	Stage 2	Median: 10 m, range: 7 m to 1 y and 1 m
Low & Manson-Bahr [86]	1922	DRC	DM	P	1	Infection	Stage 2	2 y to 5 y
Cooke et al. [36]	1930	Ghana	DM	P	1	Infection	Stage 2	<1 y and 5 m
Cates & McIlroy [94]	1950	Gambia	Inoculation	P	1	Infection	Stage 2	≥7 y
Dreyfus et al. [95]	1959	Guinea or Chad	CM	A	1	Infection	Stage 2	7 m to 3 y
Coulaud et al. [89]	1969–1975	Gabon	CM	P	2	Infection	Stage 2	6 m; 2 y to 3 y
Taelman et al. [83]	1983	DRC	CM	P	1	Infection	Stage 2	≥3 y and 1 m
Grau-Junyent et al. [96]	1986	Equatorial Guinea	CM	P	1	Infection	Stage 2	3 y
Blanchot et al. [97]	1988	Angola	CM	P	1	Infection	Stage 2	3 y and 2 m
Buissonnière et al. [98]	1989	Senegal	Serology	P	1	Infection	Stage 2	<9 m
Otte et al. [99]	1993	Cameroon	CM	P	1	Infection	Stage 2	≥2 y
Damian et al. [100]	1993	Nigeria	CM	P	1	Infection	Stage 2	3 m to 4 m
Serrano-Gonzalez et al. [101]	1995	Equatorial Guinea	CM	P	1	Infection	Stage 2	≥3 y
Kirchhoff [102]	1997	West Africa	PCR	P	1	Infection	Stage 2	≥12 y
Raffenot et al. [103]	1997	Guinea	CM	P	1	Infection	Stage 2	9 m to 1 y and 3 m
Sahlas et al. [104]	2000	DRC	CM	P	1	Infection	Stage 2	≥1 y and 3 m
Low & Manson-Bahr [86]	1922	Equatorial Guinea	DM	P	1	Infection	Stage 2/moribund	<2 y and 4 m
Bonnal et al. [105]	1962	Mali	Rx	P	1	Infection	Stage 2/moribund	4 y to 6 y
Bédat-Millet [106]	1995	DRC	CM	P	1	Infection	Stage 2/moribund	≥6 y (≥2 y to Stage 2)
Daniels [107]	1906	Uganda	DM	P	1	Infection	death	5 y
Duren & van den Branden [108]	1932	DRC	DM	P	1	Stage 1, healthy	Stage 1, ill c	2 y and 1 m
Moustardier et al. [109]	1933	Burkina Faso	DM	P	1	Stage 1, healthy	Stage 1, ill c	≥2 y and 1 m
Checchi et al. [15]	1990s–2000s	Uganda, Sudan	CM	A and P	298	Stage 1 serological suspect	Stage 2	Mean: 1 y and 5 m, median: 1 y
Grant et al. [110]	1941	Nigeria	DM	P	1	Stage 1, healthy	Stage 2	3 y
Robinson et al. [111]	1978	Nigeria	CM	P	1	Stage 1, healthy	Stage 2	≥3 y
Martin & Darré [112]	1910	Republic of Congo	DM	A	1	Stage 1, healthy	Death	4 y
Moustardier et al. [109]	1933	Various	DM	P	6	Stage 1	Stage 2	≥2 y
Blanchard & Toullec [113]	1930	Senegal	DM	P	1	Stage 1	Stage 2	≥2 y
Riou & Moyne [114]	1933	Senegal	DM	P	1	Stage 1	Stage 2	≥4 y
Sartory et al. [115]	1910s	Unknown	DM	P	1	Stage 1	Stage 2	≥8 y
Baonville et al. [116]	1920s	DRC	DM	P	1	Stage 1	Stage 2	≥10 y
Pinard et al. [117]	1939	Republic of Congo	DM	P	1	Stage 1	Stage 2	≥15 y
Collomb et al. [118]	1950s	Various	DM	P	26	Stage 1	Stage 2/moribund	Median: ≥3 y, range: ≥1 y to ≥4 y
Guérin (in Laveran [4])	1850s–1860s	Various (West African slaves)	clinical	P	?	Stage 1	Stage 2/moribund	≥5–8y
Edan [39]	1970s	Republic of Congo	CM	P	22	Start of symptoms	Stage 2	Median: 3 m, range: <3 m to 6 y
Milord et al. [40]	1987–1991	DRC	CM	Unknown	207	Start of symptoms	Stage 2	Mean: 2 y and 2 m, range: 4 m to 6 y and 6 m
Blum et al. [38]	1997–1998	Angola	CM	A and P	588	Start of symptoms	Stage 2	Median: 4 m to 6 m; >1 y in 27 patients (4.9%)
Blum et al. [37]	2000s	Various	CM	A and P	2541	Start of symptoms	Stage 2	Median: 8 m, >2 y in 62 patients (2.8%)
Greggio [25]	1911	DRC	DM	A	183	Stage 1 and 2, not too ill	Death	Median: 1 y and 2 m, range: 0 m to 7 y and 6 m
Checchi et al. [15]	1990s–2000s	Uganda, Sudan	n/a (model-based)			Stage 2	Death	Mean: 1 y and 4 m, median: 11 m

a DM  =  direct microscopy on blood, cerebrospinal fluid or gland puncture fluid; CM  =  microscopy after blood concentration; PCR  =  polymerase chain reaction; Rx  =  empirical diagnosis based on dramatic improvement post antritrypanosomal treatment.

b A  =  active community screening; C  =  convenience screening; P  =  passive case detection; V  =  vertical transmission of HAT to patient's baby.

c Excluding patients treated for symptoms associated with trypanosomal chancre in the first month after reporting a tsetse bite.

Furthermore, potential sources of bias that specifically weaken the strength of inference were ubiquitous (Table 3), and no studies was unaffected by at least one of these biases. A large proportion of studies were conducted during the years when microscopic inspection of lymph node aspirates, blood, or CSF was the sole means of diagnosis, and the taxonomy of trypanosomes was not well established. It is now known that light microscopy is a very insensitive technique [20], mainly due to the low and undulating parasitaemia characteristic of HAT. More importantly, several non-pathogenic trypanosome species, such as *T. congolense*, *T. brucei brucei (bouaflé)*, and *T. evansi*, are now known to occasionally infect humans [21],[22],[23],[24], yet these forms are essentially indistinguishable from *T. b. gambiense* by microscopy. Positive microscopy in the presence of symptoms typical of HAT would have reduced the likelihood of such species misdiagnosis, but not entirely, since several diseases, such as malaria, tuberculosis, and HIV, can mimic HAT. Whilst recognising these shortcomings, it is important to note that such occurrences are rare.

**Table 3:** Main Potential Sources of Bias, and Number of Eligible Reports Affected, by Type of Report

Possible Source Of Bias	Implications	Natural Progression (*n* = 27)	Duration of Infection (*n* = 42)	Spontaneous Clearance (*n* = 4)
Insensitive diagnosis due to microscopy on non-concentrated blood only.	Absence of infection noted during the observation period cannot be taken as proof of clearance.	23	19	4
No certainty about sub-species.	Benign infections could actually be due to transient animal trypanosomes.	25	39	4
Information on symptoms and their duration are based on patient recall.	Patients might systematically over- or under-report the duration of symptoms, or provide inaccurate data.	0	4	0
Information on time of infection based on patient recall.	Patients might not accurately report when they were last exposed to tsetse bites (e.g., patients who had left Africa might not report the most recent trip).	1	29	1
No information about traditional or other treatments during observation period.	Patients might have been cured thanks to traditional therapies or antimicrobials taken for other infections, but which may have limited activity against HAT.	27	22	3
Group of patients is highly self-selected.	Patients with mild infections are more likely to be healthy and thus refuse treatment or be included in natural progression experiments. Patients who remain healthy are more likely to be observed for longer. Measuring the duration of disease based solely on patients who have already died may result in under-estimation. Patients who attend a health centre may be unrepresentative.	6	7	1

Publication bias is also possible; for example, studies showing unusual outcomes could have been reported more frequently than those confirming the textbook pattern, and may thus make up a disproportionate fraction of the literature in our review, potentially suggesting that some phenomena are more frequent than they actually are.

### Reports of the Natural Progression of HAT

Quantifying the relative frequency of different outcomes of untreated infection would allow for the development of realistic mathematical models that (i) predict the proportion of infections that will not become symptomatic and thus will not be captured by passive case detection, (ii) explore the contribution of these non-pathogenic infections to maintaining transmission, (iii) help to optimise screening strategies that target non-pathogenic infections with the ultimate aim of eliminating foci altogether, and (iv) predict the impact of different control strategies on morbidity and mortality.

Eligible reports of the outcome of untreated HAT (Table 1) are hard to interpret, mainly because the observation periods are censored at about 3 years for the largest case series, and because the case series are a mix of early and late infections. As expected, the case fatality ratio generally increased when the duration of observation was longer, reaching 73% in a case series in the Belgian Congo that was followed for 4 and a half years [25]. Case fatality ratios were calculated based on cases known to have died, but would be higher if patients who disappeared or had an unknown endpoint are considered dead, notably for the series by Marshall and Vassallo [26] and Heckenroth [27]; indeed, in the latter study only three of 36 patients (8.3%) were known to remain healthy after 4 years of follow-up.

Two reports contradict this general trend in case fatality. In Ivory Coast, Jamonneau et al. [28],[29] observed a group of 15 parasitologically confirmed patients who refused treatment. After 4 years, at least seven were no longer parasitaemic, only one progressed to stage 2, and of the remainder, only three had clinical signs of stage 1. Six of the patients were followed for a further 2-year period, at the end of which three were mildly ill, and all were parasite-negative microscopically but PCR-positive. Genotyping using microsatellite markers revealed that two were mono-infected with a new *T*. *brucei* s.l. strain, while four had a co-infection with the new strain and *T. b. gambiense* group 1. In The Gambia, Todd [30],[31] traced 12 patients over 9 years following initial diagnosis, and found eight still healthy, of whom one was seen again 13 years later and found to be clinically well despite reportedly not receiving treatment.

This review does not include the famous “FEO” case, a Togolese female who remained parasitaemic and healthy over 23 years of follow-up, despite repeated treatment courses [32]; the strain was morphologically consistent with *T. b. gambiense*, but induced more chronic infections in animals, and may have been non-gambiense.

### Reports of Spontaneous Infection Clearance

Reports of spontaneous infection clearance are rare. Harding and Hutchinson [33] report that nine out of 75 Sierra Leonean asymptomatic parasite-positive cases at baseline no longer showed parasites in weekly tests over the next 2 months. In this community (Fuero), no significant mortality attributable to HAT was noted in spite of infection attack rates >25% over 3 years. Presenting cases were asymptomatic, with infrequent cervical gland involvement and scanty parasitaemia. Interestingly, case finding and mass chemoprophylaxis were less successful in controlling transmission in this site than in neighbouring areas where classical HAT was prevalent. The authors speculated that a novel strain could have been responsible for the atypical outbreak in Fuero.

Dyleff [34] reported that three Europeans returning from the French Congo, who were positive for HAT at an initial test, subsequently tested negative in all further tests in various laboratories.

Barlovatz [35] described a patient from the Belgian Congo who refused treatment. Three years and 3 months later, various tests, including inoculation of six Guinea pigs with his blood, were negative.

Cooke et al. [36] reported on an Englishman returning from Nigeria and Ghana who, upon post-repatriation screening, was found to be infected but mistakenly discharged without treatment; 6 months later no trypanosomes were found in blood films or upon animal inoculation. The patient was nonetheless treated as a precaution.

### Reports of the Duration of HAT Infections Progressing to Disease

The duration of pathogenic HAT infections is a critical parameter for the development of predictive mathematical models since it determines opportunities for further transmission to flies and for case detection before the onset of irreversible sequelae or death. The rates of progression from stage 1 to stage 2 from stage 2 to death are also indispensable parameters in any model of HAT transmission.

Most reports on the progression of HAT have been based on individuals developing HAT disease after exposure to tsetse bites at a known point in time. While baseline and endpoint status differs widely across these studies, the duration of *T. b. gambiense* stage 1 ranged from a few months to a few years (Table 2). Only three cases with duration beyond 8 years were found among these reports (including a patient who took at least 12 years to progress from infection to stage 2, and another for whom at least 15 years elapsed between stage 1 and 2). An unknown number of cases of advanced stage 2 were diagnosed clinically by Guérin in the Caribbean among slaves abducted from West Africa 5–8 years earlier [4]. Four of the larger series suffered from considerable selection bias: Blum et al. [37], Blum et al. [38], Edan [39], and Milord et al. [40] studied stage 2 patients coming for treatment whose duration of symptoms was probably dependent on treatment-seeking behaviour; Greggio [25] only included patients who had already died by the time of the study, thus biasing observations towards short duration cases. Nonetheless, all large series showed a left-skewed distribution of duration (unpublished data), with fewer than 5% of cases lasting more than 4 years up to a maximum of 6 to 7 years. Interestingly, Manson believed that HAT symptoms can arise up to seven years post infection [4]. Residents of Gorée Island (Senegal) also reportedly considered themselves safe from HAT if no symptoms arose in the 7 years after a trip to the mainland [4]. Fèvre et al. [18] aggregated 88 patients reviewed in Yorke [17] and 8 patients reported after diagnosis in Europe and North America (all presented here with the original citations), all of whom had a plausible time of infection and known time of death or treatment; survival analysis suggested a median duration of infection of 36 months. Checchi et al. [15] derived indirect estimates of stage 1 duration (mean 17 months, median 12 months) from survival analysis of 298 untreated serological suspects' time to progression from suspected stage 1 to confirmed stage 2, and deduced stage 2 duration (mean 16 months, median 11 months) from the observed stage 1 to 2 ratio in the community. This gives a rough estimate of 33 months for the mean total duration of infection. Subsequent adjustment for diagnostic specificity and stage misclassification, however, suggests that the mean stage 2 duration may have been overestimated, and could be closer to 10 months (Checchi et al., unpublished observations).

## Biological Considerations

### Natural Progression

The assumed 100% lethality of HAT seems surprising from an evolutionary perspective: over time, parasites are believed to regulate their virulence so as to avoid killing off their primary hosts, which for gambiense HAT are humans (an animal reservoir has been demonstrated [41], but is generally considered of marginal importance [42]). Furthermore, where HAT has been a major cause of mortality, evolutionary pressure on humans might have resulted in the development of trypano-tolerance. Among infectious agents that rely heavily on humans as a reservoir, none is known to be 100% fatal, with the possible exception of HIV, which is a relatively new disease in humans, and for which non-disease progressors are already reported [43]. This classical paradigm of evolutionary drift towards non-virulence has been challenged, especially for vector-borne and other indirectly transmitted pathogens, which tend to be more virulent than directly transmitted pathogens, and may require high virulence to debilitate hosts, potentially exposing them to greater transmission, for example through more intense vector bites [44]. However, HAT does not fit this pattern: HAT patients' prostration actually reduces their contact with tsetse colonies around watering points or cultivation areas. Furthermore, much mortality from infections is in fact caused by host immunopathology, which may not be an intended outcome for the parasite [45]. HAT may fit this scenario: death occurs as a result of inflammatory processes, rather than damage from parasite exploitation of the host's tissues.

*T. b. gambiense* survives the haemolymphatic immune response through an underlying resistance to lytic factors in human serum, including apolipoprotein L1 (non-human infectious trypanosome species do not have this ability) [46]. They also resist the acquired immune response through a process of surface antigenic variation whereby new antigenic sub-populations constantly arise, thus escaping antibody-mediated immunity [47]. The repertoire of variant surface glycoprotein (VSG) genes runs into the order of a thousand, and the potential repertoire grows as genetic recombination ensures the genes mutate and evolve even within the course of an infection. One could speculate that any HAT infection would eventually disappear when this repertoire is exhausted, as is the case for *Borrelia spp.*, the causative agent of relapsing fever. However, current uncertainty about rates of VSG switching, which range from >1 per 10^3^ to <1 per 10^6^ parasite replications [48], makes it difficult to predict when an infection might deplete its repertoire of antigens and therefore how long any chronic carriage might last relative to the host's lifespan. To our knowledge, no other extracellular microparasitic infections based in the haemolymphatic system are known to survive long periods in the human host without colonising specific tissues. The existence of intracellular forms of *T. brucei* has been proposed: this suggestion rests on weak evidence, but has not formally been disproved [49].

How else could spontaneous parasite clearance occur? When specific immunity to the currently expressed VSG is at its peak, parasite density hits a trough, before recovering as a new VSG population emerges. Even at its lowest level, total parasite numbers probably remain in the thousands (assuming a minimum parasitaemia of 10/mL and 5 L of blood), and it is therefore unlikely that the infection would be extinguished due to chance. Some strains, however, might feature an abnormally slow VSG switching rate, allowing host immunity to eradicate the current antigenic sub-population before the next one takes over. Alternatively, certain strains might not express genes coding for resistance to human serum. Unlike in *T. b. rhodesiense*, serum resistance is believed to be a stable trait in *T. b. gambiense*, but exceptions might occur. However, one might expect such strains to go extinct due to selection pressure, and therefore to not feature prominently among those circulating at any given time.

An alternative hypothesis is that some humans carry trypano-tolerance traits, as noted already in various animal species in whom trypanosomes do not necessarily lead to death [50],[51]. It is also possible that certain human sub-populations might be deficient in genes encoding lytic factors for non-gambiense trypanosomes (such as *T. congolense* or *T. brucei brucei*), which might survive briefly in the blood, cause only mild illness, and be mistaken for gambiense. Indeed, a recently reported case of *T. evansi* infecting a man in India involved a patient deficient in apolipoprotein L1 [52].

Chronic carriage, on the other hand, would presumably occur if the host cannot overcome the haemolymphatic infection, but the parasites do not cross the BBB. *T. b. gambiense* itself may modify the permeability of the BBB, either directly through signalling and endothelial cell apoptotic pathways, or indirectly due to immunopathogenic effects of infection in the haemolymphatic system [53]. Parasites initially invade the central nervous system (CNS) through vulnerable points such as the choroid plexus and the thalamus. CNS invasion may carry mixed evolutionary advantages for the parasite, as it allows escape from host immunity, but also leads to earlier host death and thus shorter duration of infectiousness.

Possible mechanisms for chronic carriage include (i) failure of given strains to modify BBB permeability, for example because they do not express certain factors (e.g., proteases believed to be required for BBB entry [54]); (ii) strains reproducing at such a slow rate that the parasite density remains extremely low, such that any CNS invasion is limited or benign; and (iii) humans with trypanosome-impermeable BBB. To our knowledge, empirical evidence supporting any of these models has yet to be obtained.

### Variability in Duration of Infectiousness

The time over which patients remain infectious, at least in pathogenic cases, is determined by how fast stages 1 and 2 progress. Variability in these parameters could be influenced by a number of parameters, including differences in strain virulence, host immunity, and host BBB permeability.

**Figure 2:**
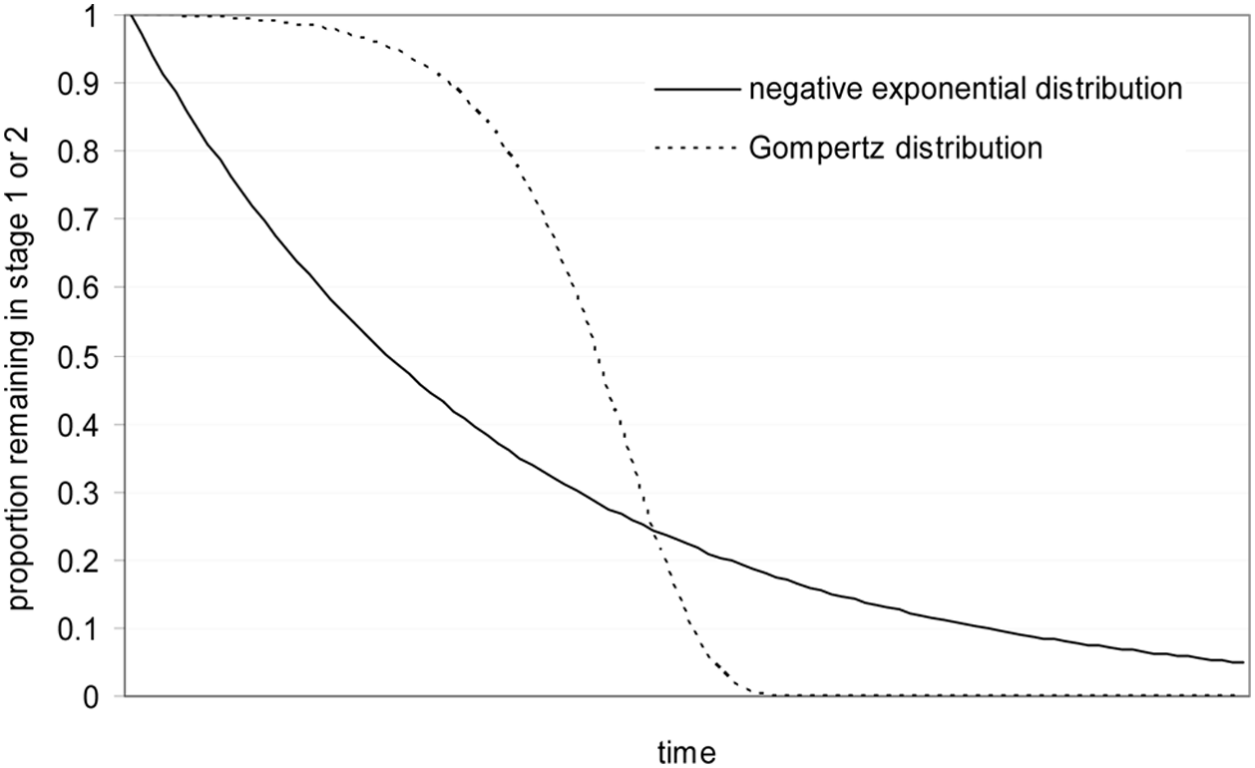
Examples of Negative Exponential and Gompertz (Non-Constant Hazard) Distributions of Survival in HAT Stages 1 or 2.

What is the typical rate of progression from stage 1 to stage 2 in pathogenic infections that do cross the BBB? Assuming that BBB modification due to HAT infection within any individual is a continuous process, and given that parasite density in the haemolymphatic system is known to undulate around a roughly constant average, the risk of CNS penetration per unit time, i.e., progression from stage 1 to stage 2, should be constant, and proportional to parasite density (obviously, differences in virulence across strains will create variability in this risk among individual infections). A constant risk of stage 2 progression would result in a negative exponential distribution of the duration of stage 1 (see Figure 2 for an illustration). Such a distribution would feature a relatively short median duration, but also comprise a tail of very long durations—in other words, “exceptions” would be expected. Indeed, this exponential decay hypothesis is corroborated by survival analysis of untreated serological suspects [15]. Phenotypic changes in the parasite population occurring at later phases of the infection, such as improved motility, would cause the risk of progression to vary with post-infection time, but to our knowledge these have not been observed.

The death rate in stage 2 is another key parameter. Once the CNS is invaded, poorly understood pathogenic processes such as macrophage activation and a complex cytokine cascade appear to be responsible for the onset of neurological symptoms [55]. However, the fact that drugs can halt and partially reverse stage 2 neurological deterioration suggests that this is a cumulative process due to parasite persistence in the CNS. Stage 2 patients are known to deteriorate progressively, suggesting that the death rate would increase with time since onset of stage 2; this time-dependence would yield a Gompertz distribution of survival in stage 2 (Figure 2), featuring very little variability in time to death, and precluding exceptional observations. Greggio [25], however, described a negative exponential survival of untreated patients.

## Discussion

### Does Human Trypano-Tolerance Exist?

Published data on the natural progression of HAT are inconclusive. Based on literature and biological considerations, we believe the following cautious conclusions can be drawn:

1. The majority of untreated gambiense HAT infections progress to death.
2. There is some evidence that patients can spontaneously recover from stage 1 HAT infection. However, all such reports are from West Africa, and may in fact involve strains other than *T. b. gambiense* group 1.
3. There is no evidence that chronic carriage exists, although it is biologically plausible. It is extremely difficult to study this outcome given current diagnostic sensitivity and the ethical requirement to treat all detected infections.
4. If spontaneous cure or chronic carriage do occur in stage 1, they probably constitute a minority of infections.
5. There is no evidence for spontaneous cure from stage 2.
6. Infections either progress to stage 2 within a few years, or not at all (as suggested by the absence of reports of very long stage 1 infection in patients who have been away from HAT-endemic areas for a known period).
7. Durations of infection beyond 6–7 years are extremely rare.

The existence of human trypano-tolerance is difficult to demonstrate formally, although it may be postulated based on its occurrence in various animal species. The two methods of detecting HAT cases, active and passive screening, do not allow observation of the natural progression of infection, since cases are treated upon laboratory confirmation, irrespective of symptoms, as stated above. Similarly, any cases who self-resolve would go unrecognised. However, it may be possible in selected sites to identify retrospectively any patients who absconded before treatment, and verify their status.

Currently, trypanosome classification relies on a rather outdated isoenzyme profile analysis [56] that may not capture the full genotypic and phenotypic variability of strains. Better characterisation of representative samples of strains circulating in various foci could be attempted using the latest genotyping techniques and improved methods of trypanosome propagation [57],[58]. In the Ivory Coast, where more modern and specific genotyping techniques such as PCR on satellite markers have been attempted, non-gambiense trypanosomes have been shown to infect humans [29],[59],[60], suggesting that elsewhere, the range of human-infective trypanosomes could be more varied than assumed. Geographic differences in apparent clinical severity of HAT infections have been reported for both *T. b. gambiense* [8],[61],[62] and *T. b. rhodesiense* [63], and may well be due to variations in parasite strain and species.

The use of PCR has also demonstrated low-level trypanosome infections in a proportion of sero-positive but parasitologically negative cases [64],[65],[66]: further study of sero-positive, parasitologically negative individuals through PCR or other advanced techniques is warranted, as this host sub-population could include chronic carriers with parasite densities below the detection threshold of currently used field diagnostic tests.

The duration of infectiousness among pathogenic cases is equally difficult to study. However, some insight about the distribution of time to symptom onset (a proportion of the total infectious period) could arise from a review of HAT cases diagnosed outside Africa over the past decades (we have attempted to locate all such published reports, but unpublished cases may exist). Further insight could be gained from asking HAT patients to recall the time of occurrence of infectious bite chancres (however, this is an uncommon sign in African patients and may be a feature of particularly virulent infections).

### Implications for Control

The epidemiological implications of the different scenarios considered for the natural history of HAT infection are outlined in Table 4. While self-resolving infections would probably be of limited epidemiological importance, chronic carriers might play a key role in perpetuating transmission. Indeed, their existence could explain how certain HAT foci appear to be extinguished, only to re-awaken mysteriously after several years, or how the chain of transmission in certain small communities appears to be sustained by only a handful of cases [67]. Preliminary results from stochastic modelling (F. Checchi, J. Filipe, D. Chandramohan, unpublished data) suggest that even a small proportion of chronic carriers would considerably decrease the chance of repeated active case detection campaigns detecting a sufficiently large number of infections to interrupt transmission; more importantly, this chance would be far lower if, as is plausible, chronic infections were harder to detect by current diagnostic tools. There is debate about the relative harms and benefits of treating all serological positives: in most prevalence scenarios, this results in a positive predictive value below 30% [68],[69], meaning a considerable number of people are needlessly treated with toxic drugs; however, such a strategy could detect most trypano-tolerant infections, and might be warranted as a one-off “catch-all” intervention when elimination of a focus appears close.

**Table 4:** Possible Scenarios for Trypano-Tolerance, and Their Likely Implications for Control Strategies

Occurrence of Trypano-Tolerant Cases	Nature of Trypano-Tolerance	
	Self-Resolving Infection	Mostly Asymptomatic or Mildly Symptomatic Chronic Carriage
Frequent	Might never be detected through passive screening, so would need to be detected actively; their contribution to transmission would depend on their infectiousness and on the average duration of infection before self-resolution.	Mass screening-based control would be imperative if chronic carriers have a significant level of infectiousness: its frequency, coverage and sensitivity would have to be very high to eliminate transmission.
Rare	Minimal influence on reproductive ratio, transmission could perhaps be interrupted through intensive passive screening even if these cases remain untreated.	Small influence on reproductive ratio, but, if not detected actively, could be responsible for perpetuating transmission even in settings with very intensive mass screening-based control.

If chronic carriage is due to an inherited parasite trait, one might expect screening to select for strains that permit chronic carriage, and ultimately for non-pathogenic clones to dominate the ecology of circulating strains, i.e., transmission would continue but the disease burden would be reduced. This is probably not borne out by field observations, as the HAT epidemics resulting from breakdown of control in the 1970s and 1980s have featured typically lethal infections. The true ecology of *T. b. gambiense* strains is probably far more complex, and regulated by host–vector–parasite interactions as well as the sharing of genetic material among strains.

The minority of long-duration pathogenic infections corresponding to the tail of the distribution of natural durations could also escape control and seed a new epidemic, especially if the end of a screening programme is decided based on a target prevalence (e.g., <0.5%).

If the HAT transmission chain is to be broken, active case detection with treatment irrespective of symptoms, along with continued surveillance, remain paramount, regardless of whether chronic carriers exist [70]. If the existence of a trypano-tolerant infection reservoir were demonstrated, case detection strategies could be adapted, for example by introducing systematic serological treatment (see above) and ensuring very high screening coverage (based on modelling work previously published by others [71],[72] and currently being done in our group, it is likely that elimination would not require detecting all chronic carriers). New safe and easily administered (i.e., oral) drugs would also help to maximise treatment coverage. Such strategies may maximise the chances of eliminating the remaining HAT foci. There is currently a window of opportunity for doing so, as many of the foci are at their lowest level of activity [73],[74] since the start of the post-colonial period, and there is renewed commitment from the World Health Organization and other agencies to pursue elimination [75].

## Supporting Information

Alternative Language Abstract S1 Translation of the Abstract into French by Francesco Checchi
(0.03 MB DOC)
