# Supplementary material for: The Natural Progression of Gambiense Sleeping Sickness: What Is the Evidence?
Source: PLoS Negl Trop Dis. 2008 Dec 23;2(12):e303. doi: 10.1371/journal.pntd.0000303 (PMC2602732; doi:10.1371/journal.pntd.0000303)
Supplement: Alternative Language Abstract S1 — Translation of the Abstract into French by Francesco Checchi (0.03 MB DOC) [file pntd.0000303.s001.doc]

**Abstract**

**Introduction**

La trypanosomiase humaine Africaine (THA, maladie du sommeil) à *Trypanosoma brucei gambiense* est généralement supposée être pathogénique et létale à 100%. Il existe néanmoins plusieurs observations qui contredisent cette hypothèse. L’existence d’infections humaines trypano-tolérantes a également été évoquée, tandis que la vraie durée des deux phases de l’infection reste mal décrite, en particulier en ce qui concerne la durée de la période infectieuse. Une meilleure compréhension de ces paramètres semble essentiale afin d’optimiser les stratégies de contrôles de la THA qui posent sur la détection des cas.

**Méthodes et résultats principaux**

Nous avons pris en considération l’existence de la trypano-tolérance humaine, et son importance potentielle, et également investigué la durée de la période infectieuse, à travers une revue de la littérature sur la progression naturelle de la THA gambiense en absence de traitement, et en prenant compte quelques considérations biologiques.

**Conclusions/Importance**

Les observations publiées suggèrent que la plupart des cas de THA gambiense sont en effet létales en absence de traitement. Les infections asymptomatiques, chroniques ou de courte durée, sont probablement minoritaires, si elles existent. La possibilité de porteurs chroniques mérite néanmoins plus de recherche : elle pourrait bien constituer le germe de nouvelles épidémies dans la suite de l’interruption des programmes de lutte.
